# Systematic selection of best performing mathematical models for in vitro gas production using machine learning across diverse feeds

**DOI:** 10.1038/s41598-025-15101-w

**Published:** 2025-08-21

**Authors:** Hamed Ahmadi, Natascha Titze, Katharina Wild, Markus Rodehutscord

**Affiliations:** Institute of Animal Science, University of Hohenheim, Stuttgart, Germany

**Keywords:** Gas production, Mathematical models, Model selection, Machine learning, Data integration, Machine learning, Animal physiology

## Abstract

In vitro gas production (GP) is commonly used to evaluate ruminant feed, yet its accurate interpretation requires robust mathematical modeling. This study systematically explores a wide array of nonlinear models to explain GP dynamics across various feed types, addressing the question: how can efficient and versatile models that accurately represent GP profiles be identified? We hypothesized that distinct feed types exhibit unique GP characteristics, effectively captured by specific models, and that statistical and machine learning methodologies can streamline model selection. Utilizing a comprehensive dataset derived from 849 unique GP profiles across concentrate feed categories—including cereal and leguminous grains and processed protein feeds—21 candidate models were rigorously evaluated based on their goodness-of-fit metrics, with a particular emphasis on Bayesian Information Criterion (BIC) for model selection. A group of three models—namely Burr XII, Inverse paralogistic, and Log-logistic—consistently emerged as top performers, demonstrating high generalizability and predictive power across feed types. Notably, our analysis indicated that model type significantly influenced GP predictions, surpassing the impact of feed type characteristics. This research establishes a decision-making framework for model selection and sets the stage for further investigations linking in vitro GP parameters to in vivo digestibility, ultimately enhancing ruminant nutrition strategies.

## Introduction

In vitro gas production (GP) is a key laboratory technique used to assess the fermentation characteristics of feedstuffs for ruminants in a controlled setting^1–3^. By simulating the digestive process primarily in the forestomach, this method provides information on the nutritional value and digestibility of various feeds, which is crucial for optimizing animal feed formulations. The GP technique involves incubating feed samples in sealed vessels with a rumen fluid-buffer mixture and measuring the volume of gas produced over time, offering a proxy for feed fermentation dynamics. Accurate modeling of GP curves is critical for interpreting fermentation data and understanding the underlying dynamics of feed digestion. Effective models can offer detailed understanding of the rate and extent of GP, which are essential for evaluating the feed. However, the complexity of the fermentation process and the variability among feed types present challenges in model selection^2^. Previous modeling efforts in GP research have explored various models, ranging from simple two-parameter models such as the Exponential to more complex ones like the four-parameter Richards, Gompertz, and Mitscherlich model^4–6^. While these models capture the basic dynamics of GP, they differ in their ability and flexibility to represent the full range of GP behavior observed across diverse feed types. Building on these foundations, it appears that a broader array of models remains unexplored and untested under a unified evaluation framework.

In the context of curve modeling, phenomenological (or empirical) models make an important contribution^7^. Unlike mechanistic models, which aim to represent the detailed biological processes driving GP, phenomenological models focus on capturing the observable patterns in GP data. These models are particularly valuable when the underlying biological mechanisms are complex or not fully understood. By fitting these models to data, the general shape and behavior of GP curves can effectively be described without needing to explicitly model the intricate fermentation processes. Despite their empirical nature, phenomenological models can sometimes be interpreted in a mechanistic context^5^. For instance, the parameters of these models may correspond to biological processes, such as the rate of fermentation or the total fermentable substrate available. This allows for a simplified mechanistic interpretation, where the empirical model approximates the underlying processes. Moreover, in situations where full mechanistic modeling is impractical, phenomenological models can serve as substitutes to generate predictions and derive insights from experimental data^7^.

This study addresses the question of how to identify efficient, meaningful, and versatile mathematical models for describing GP across various feed types. We hypothesized that distinct feed types exhibit unique GP profiles that can be accurately captured by specific nonlinear models, and that statistical analysis combined with machine learning will identify effective models for these patterns, with a few consistently performing well across feed types to simplify model selection. The objectives were to systematically explore a broad array of mathematical models, including many phenomenological ones, evaluate their ability to fit in vitro GP data from different feed types, and use statistical post-hoc analysis alongside machine learning methods to pinpoint the best-performing models, ultimately creating a decision-making framework for efficient model selection of GP kinetics.

## Materials and methods

The data used in this study were obtained from previously published references. Therefore, no new animal experiments were conducted, and approval for animal experiments was not required.

### Description of the dataset

The dataset utilized in this study consists of GP data derived from a series of experiments designed to assess various feed types^8–15^. The experiments were conducted with single feed ingredients and mixed compound feeds.

***Single feed ingredients***: Seven distinct feed ingredients were evaluated individually, including: Barley (20 genotypes), Corn (20 genotypes), Lupin grains (12 variants, differing by genotype, harvest year, and location), Pea grains (13 genotypes), Rye (20 genotypes), Triticale (20 genotypes), and Wheat (20 genotypes).

***Compound feeds***: In these trials, samples of single feed ingredients were tested alone or mixed in different combinations. Some of them were tested in different forms, such as mash or pellets with varying protein levels. The following feed types were included in the compound feed trials: Barley, Compound feed, mash form (8 levels of protein), Compound feed, pellet form (8 levels of protein), Corn, Corn gluten, Distiller’s dried grains with solubles (DDGS), Faba beans, Full-fat soybeans, Rapeseed meal, Soybean meal, Sugar beet pulp, Sunflower meal, Wheat, and Wheat bran.

The trials were conducted using the Hohenheim Gas Test (HGT) with rumen fluid from cows, following the standardized approach described by^1^with modifications outlined in^8^. The GP (mL/200 mg dry matter) was recorded at nine time points: 0, 2, 4, 6, 8, 12, 24, 48, and 72 h. This time-course data offers a detailed view of GP dynamics over the specified period. Each feed was replicated in the assay between 3 and 7 times due to the specific experimental setup for each feed type, resulting in a total of 849 unique curves for GP estimation.

To simplify the analysis and reduce the dimensionality of the dataset, feeds were categorized into six groups based on their common characteristics. This categorization, referred to as the “six feed categories,” helped in subsequent mathematical and statistical analyses, allowing for comparisons across different feed types and insights into model performance relative to each feed. The six feed categories were: (1) Compound; includes mixed feeds in mash and pellet forms, (2) Corn, (3) Processed protein; includes corn gluten, DDGS, rapeseed meal, soybean meal, and sunflower meal, (4) Legumes; includes faba beans, full-fat soybeans, lupin grains, and pea grains, (5) Soft cereal; includes barley, rye, triticale, and wheat, and (6) Others; includes sugar beet pulp and wheat bran.

**Fig. 1:**
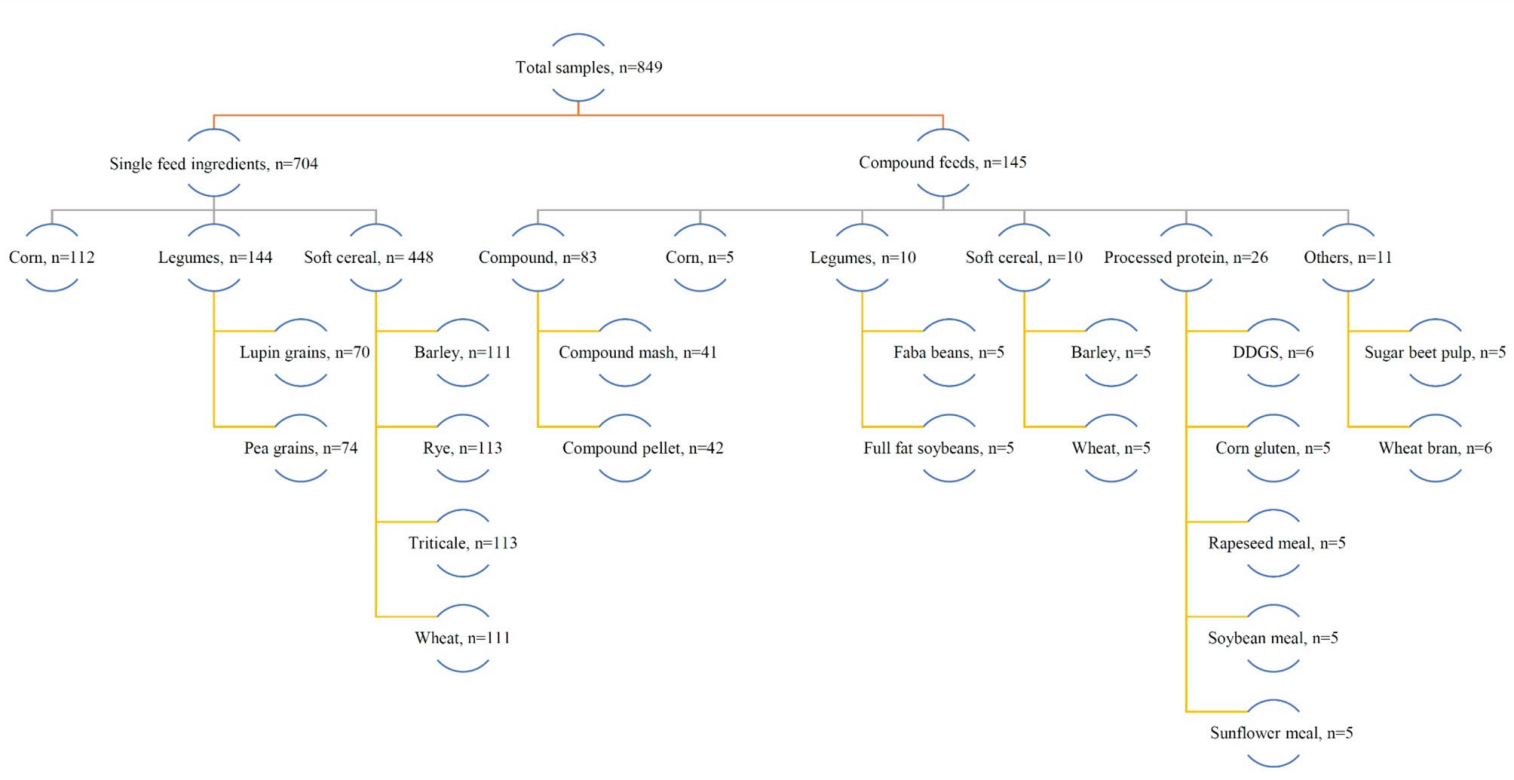
Overview of feed types and sample sizes for model fitting.

**Fig. 2:**
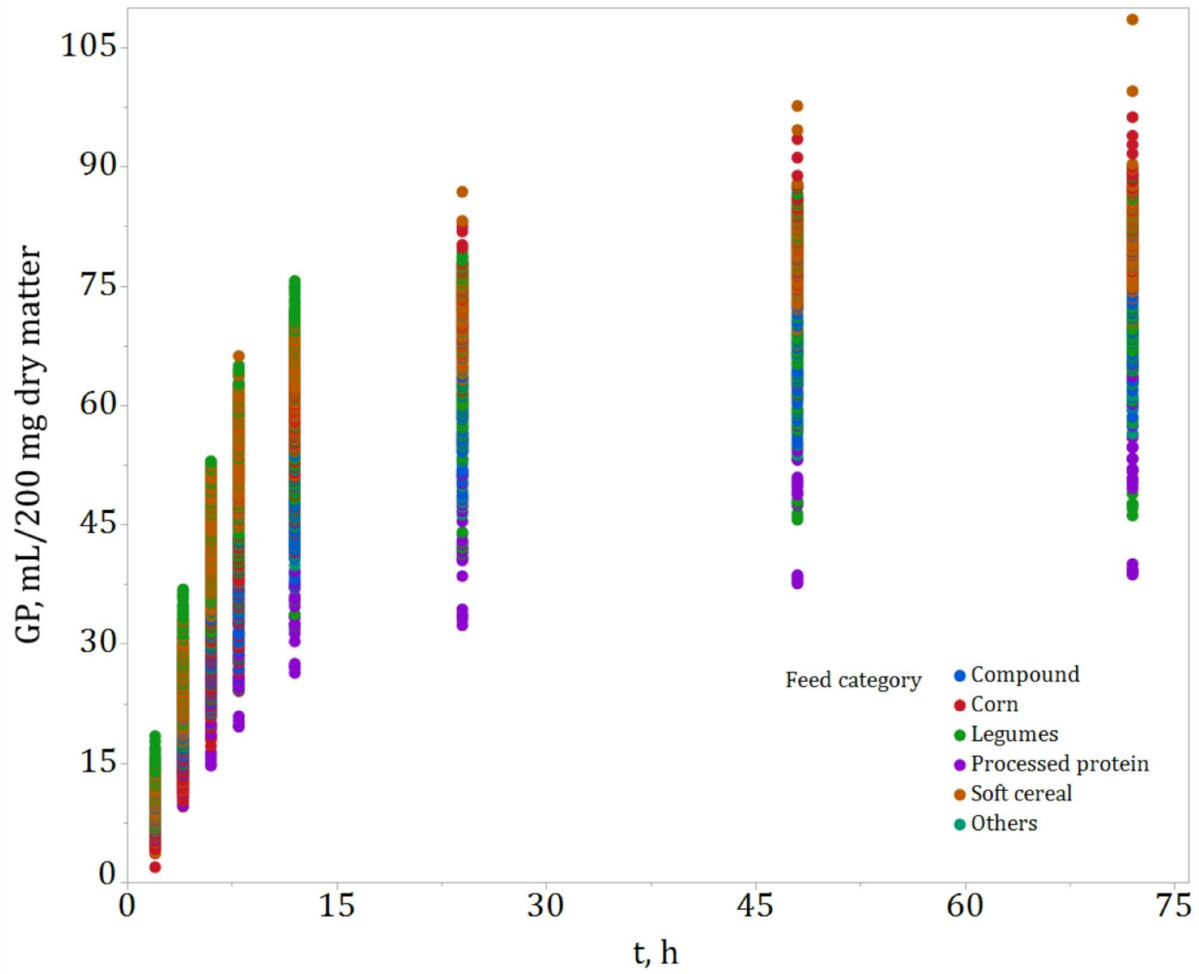
In vitro gas production (GP) profiles over time (t) (*n* = 849) grouped by feed category. This figure presents the raw GP data from all individual profiles. Since each profile was later fitted with 21 models (resulting in 17829 fits), inclusion of fitted curves or confidence intervals would obscure the data and reduce clarity; thus, they are not shown.

Figure 1 summarizes the dataset’s structure, including the experimental setup, feed types and categories, and sample sizes. The total of 849 estimated GP profiles grouped by each feed category is illustrated in Fig. 2.

### Mathematical model exploration and fitting

#### Understanding the basics of the GP curve

**Fig. 3:**
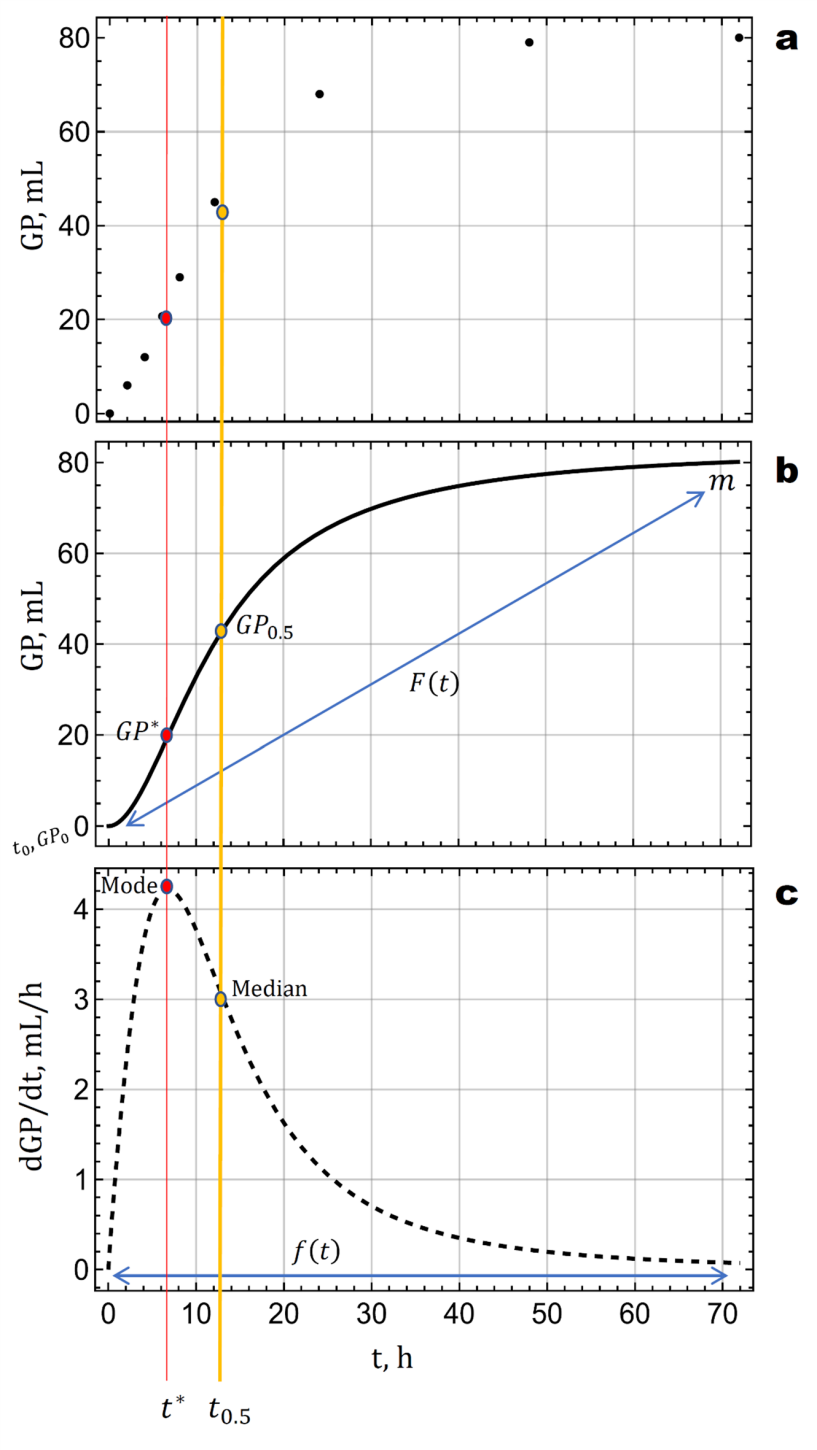
Scheme of the basics of the in vitro gas production (GP) curve. (**a**) A typical plot of cumulative GP, measured in mL per 200 mg dry matter, over time ($\:t$, in hours). (**b**) A curve approximated by $\:F\left(t\right)$ which is a mathematical model used to smooth and fit the cumulative GP data from plot **a**. This fitted curve starts at the initial GP ($\:{GP}_{0}$) at time $\:{t}_{0}$ and monotonically increases toward the potentially maximum GP (i.e. $\:m$). (**c**) A rate of GP (RGP) approximated by $\:f\left(t\right)$, which is the first derivative of the GP curve, $\:\frac{dGP}{dt}$. This derivative curve represents how the rate of change in GP evolves over time. In this context, $\:F\left(t\right)$ denotes the cumulative distribution function (CDF) that approximates the cumulative GP, while $\:f\left(t\right)$ represents the probability density function (PDF) and is equivalent to $\:{F}^{{\prime\:}}\left(t\right)$. Key points on the curve include: $\:{GP}_{0}$ is the starting value of GP at time $\:{t}_{0}$ (by definition, $\:{GP}_{0}=0$ in reality. However, some models may predict a non-zero $\:{GP}_{0}$, and this discrepancy arises from the structure of the models); $\:m$, is the asymptotic value that GP approaches as time progresses indefinitely; Inflection point ($\:{t}^{*}$), the time when the rate of GP reaches its maximum and the curve changes concavity, marking a critical phase in the GP process, with the corresponding GP amount denoted as $\:{GP}^{*}$; and Half-life ($\:{t}_{0.5}$), the time at which half of the maximum GP ($\:{GP}_{0.5}=\frac{m}{2}$) is achieved. The points $\:{t}^{*}$ and $\:{t}_{0.5}$ represent the mode and median of the PDF $\:f\left(t\right)$, respectively, and can be analytically obtained. This figure is not based on fitted data or statistical inference and is intended solely for explanatory purposes.

The GP profiles exhibited considerable variability in their shapes. Depending on feed types, some profiles demonstrated rapid and steep increases, while others displayed more gradual, S-shaped, or sigmoidal curves. The ideal model must accommodate and accurately describe these diverse dynamics^5^. Understanding certain key terminology and model concepts specific to our context is essential. These concepts are integral to the analysis presented in this study, and are relevant to interpreting the subsequent findings and discussions. To accurately capture and model the dynamics of GP over time, we developed a comprehensive mathematical framework based on phenomenological modeling, grounded in the principles of statistical distribution functions. Our framework centers on the two fundamental statistical notions of cumulative distribution function (CDF) and the probability density function (PDF). The CDF, denoted as $\:F\left(t\right)$, represents the cumulative amount of gas produced over time, obtained by integrating the PDF. This process offers a comprehensive view of the total gas produced, starting from the initial GP ($\:{GP}_{0}$) at time $\:{t}_{0}$ and monotonically increasing toward the asymptotic maximum GP ($\:m$), reflecting the total yield as time progresses. Fitting the CDF to observed data is critical in smoothing and modeling the cumulative GP profile. The PDF, denoted as $\:f\left(t\right)$, is the first derivative of the CDF and represents the rate of change of GP (RGP) over time, highlighting periods of rapid increase, peak production, and subsequent decline. Using both the CDF and PDF is considered essential for capturing the varying behavior of different GP profiles. Additionally, certain key points help further clarify the dynamics of the GP process (Fig. 3).

##### Inflection point ($\:{\varvec{t}}^{\varvec{*}}$)

This is the time at which the rate of GP reaches its maximum and the curvature of the GP curve changes. At this point, the GP rate shifts from accelerating to decelerating, indicating a critical phase in the production process. The inflection point, $\:{t}^{*}$, is where the RGP curve’s concavity changes, and it is associated with the maximum rate of GP, $\:{GP}^{*}$. This parameter is crucial for understanding the peak phase of RGP, as it highlights when the system transitions from its most vigorous gas-generating period to a more stable or slowing phase. Identifying $\:{t}^{*}$ helps in assessing the efficiency and peak performance of the feed’s fermentative process^16^.

##### Half-life ($\:{\varvec{t}}_{0.5}$)

This represents the time required for the cumulative GP to reach half of its maximum value, denoted as $\:{GP}_{0.5}$ (which equals $\:\frac{m}{2}$). The half-life, $\:{t}_{0.5}$, is a measure of the median time needed to achieve half of the total potential GP. This parameter is essential for understanding the speed and efficiency of the fermentation process. A shorter half-life indicates a quicker attainment of significant GP, reflecting more rapid fermentation kinetics, while a longer half-life suggests a slower process. Thus, $\:{t}_{0.5}\:$reflects the temporal dynamics of GP and the effectiveness of the feed in generating gas over time^16^.

#### Model inclusion and selection criteria

To identify models capable of effectively describing GP profiles, we employed a structured two-step framework.

In the first step, we established basic conditions for model inclusion, focusing on both statistical and mathematical criteria. Specifically, the model $\:F\left(t\right)$ must be the CDF of a recognized PDF $\:f\left(t\right)$. We also imposed a constraint that $\:f\left(t\right)$ should have no more than three parameters to maintain simplicity. Furthermore, we required the model to exhibit certain shape characteristics, such as a unimodal mode if present, non-negativity from the initial time $\:{t}_{0}\:$to infinity $\:{t}_{{\infty\:}}$, an asymptotic approach to zero as $\:t$ tends to $\:{t}_{{\infty\:}}$, and a positively skewed distribution with a longer right tail. Additionally, and most importantly, the model needed to have explicit solutions for the mode and median of $\:f\left(t\right)$ to ensure practical applicability in the analysis.

In the second step, we added further conditions for practical data fitting. We ensured that the integration of $\:f\left(t\right)$ from $\:{t}_{0}$ to $\:{t}_{{\infty\:}}$, when multiplied by the maximum GP ($\:m$), resulted in a CDF $\:F\left(t\right)$ with specific properties: non-negativity, a monotonic increase over time until it stabilizes, an approach towards $\:m$ as $\:t$ approaches infinity, and straightforward calculation of the inflection point. Additionally, the final model $\:m\times\:F\left(t\right)$ was required to have four or fewer parameters to maintain manageability and interpretability^5,7^.

We conducted an extensive literature review across diverse scientific fields to find suitable models meeting these criteria. Initially, 86 models were identified that met the basic inclusion conditions. After refining the list and consolidating models with different names but identical structures, we narrowed it down to 63 distinct models for further evaluation. In the second selection step, we tested all 63 models on GP data from all feeds. Models that failed to fit even a single GP profile were discarded. “Failed to fit” is defined as cases where the fitting procedure did not converge, NaN parameter estimates were returned, or biologically implausible outputs such as negative predictions were produced^4^. We also excluded models with consistently non-significant parameters to ensure that each remaining model had a meaningful description of GP dynamics. Additionally, we confirmed that models with more than two parameters provided unbiased predictions by analyzing the predicted versus residual plots, which showed that the residuals were randomly distributed without any systematic patterns^17^. Through this rigorous selection process, we ultimately identified 21 models that met all the criteria and were deemed suitable for further evaluation in our study. Details of these models are presented in Table 1.

**Table 1:** Models explored for fitting to in vitro gas production (GP) data^1^.

Model number	Model name	$\:\varvec{F}\left(\varvec{t}\right)$	$\:{\varvec{F}}^{\varvec{{\prime\:}}}\left(\varvec{t}\right)$	$\:{\varvec{t}}^{\varvec{*}}$	$\:{\varvec{t}}_{0.5}$	Parameter assumption^2^	Domain	Reference^3^
1	Burr III	$\:m{\left(1+{t}^{-a}\right)}^{-p}$	$\:\frac{map{\left(1+{t}^{-a}\right)}^{-p}}{\left(1+{t}^{a}\right)t}$	$\:{\left(\frac{ap-1}{1+a}\right)}^{1/a}$	$\:{\left({2}^{1/p}-1\right)}^{-1/a}$	$\:a,p>1$	$\:t\ge\:0$	^26^
2	Burr XII	$\:m-m{\left(1+{\left(rt\right)}^{a}\right)}^{-p}$	$\:\frac{map{\left(rt\right)}^{a}}{{\left(1+{\left(rt\right)}^{a}\right)}^{p+1}t}$	$\:\frac{{\left(\frac{a-1}{1+ap}\right)}^{1/a}}{r}$	$\:\frac{{\left({2}^{1/p}-1\right)}^{1/a}}{r}$	$\:p>0;\:a>1$	$\:t\ge\:0$	^26^
3	Cauchy	$\:\frac{m\left(\pi\:+2\text{A}\text{r}\text{c}\text{T}\text{a}\text{n}\left[r\left(t-\mu\:\right)\right]\right)}{2\pi\:}$	$\:\frac{mr}{\pi\:\left(1+{r}^{2}{\left(t-\mu\:\right)}^{2}\right)}$	$\:\mu\:$	$\:\mu\:$		$\:t\in\:\left(-\infty\:,\infty\:\right)$	^32^
4	Dagum	$\:m{\left(1+{\left(rt\right)}^{-a}\right)}^{-p}$	$\:\frac{map{\left(rt\right)}^{ap}}{{\left(1+{\left(rt\right)}^{a}\right)}^{p+1}t}$	$\:\frac{{\left(\frac{ap-1}{a+1}\right)}^{1/a}}{r}$	$\:\frac{{\left({2}^{1/p}-1\right)}^{-1/a}}{r}$	$\:a,p>1$	$\:t\ge\:0$	^33^
5	Exponential 2p	$\:m\left(1-{e}^{-rt}\right)$	$\:mr{e}^{-rt}$	$\:0$	$\:\frac{\text{L}\text{o}\text{g}\left[2\right]}{r}$		$\:t\ge\:0$	^34^
6	Exponential 3p	$\:m\left(1-{e}^{-r\left(t-\mu\:\right)}\right)$	$\:mr{e}^{-r\left(t-\mu\:\right)}$	$\:\mu\:$	$\:\mu\:+\frac{\text{L}\text{o}\text{g}\left[2\right]}{r}$		$\:t\ge\:\mu\:$	^34^
7	Exponentiated exponential	$\:m{\left(1-{e}^{-rt}\right)}^{a}$	$\:\frac{mar{\left(1-{e}^{-rt}\right)}^{a}}{{e}^{rt}-1}$	$\:\frac{\text{L}\text{o}\text{g}\left[a\right]}{r}$	$\:-\frac{\text{L}\text{o}\text{g}\left[1-{2}^{-1/a}\right]}{r}$	$\:a>0$	$\:t\ge\:0$	^35^
8	Frechet	$\:m{e}^{-{\left(rt\right)}^{-a}}$	$\:\frac{ma{e}^{-{\left(rt\right)}^{-a}}}{{r}^{a}{t}^{1+a}}$	$\:\frac{{\left(\frac{a}{1+a}\right)}^{1/a}}{r}$	$\:\frac{{\text{L}\text{o}\text{g}\left[2\right]}^{-1/a}}{r}$	$\:a>0$	$\:t\ge\:0$	^32^
9	Generalized logistic	$\:m-\frac{m}{{\left(1+{e}^{t}\right)}^{a}}$	$\:\frac{ma{e}^{t}}{{\left(1+{e}^{t}\right)}^{a+1}}$	$\:-\text{L}\text{o}\text{g}\left[a\right]$	$\:\text{L}\text{o}\text{g}\left[{2}^{1/a}-1\right]$	$\:a>0$	$\:t\in\:\left(-\infty\:,\infty\:\right)$	^36^
10	Gompertz	$\:m\left(1-{e}^{\frac{r-r{a}^{t}}{\text{L}\text{o}\text{g}\left[a\right]}}\right)$	$\:mr{a}^{t}{e}^{\frac{r-r{a}^{t}}{\text{L}\text{o}\text{g}\left[a\right]}}$	$\:\frac{\text{L}\text{o}\text{g}\left[\frac{\text{L}\text{o}\text{g}\left[a\right]}{r}\right]}{\text{L}\text{o}\text{g}\left[a\right]}$	$\:\frac{\text{L}\text{o}\text{g}\left[1+\frac{\text{L}\text{o}\text{g}\left[2\right]\text{L}\text{o}\text{g}\left[a\right]}{r}\right]}{\text{L}\text{o}\text{g}\left[a\right]}$	$\:a>1$	$\:t\ge\:0$	^37^
11	Gumbel	$\:m-m{e}^{-{e}^{r\left(t-\mu\:\right)}}$	$\:mr{e}^{r\left(t-\mu\:\right)-{e}^{r\left(t-\mu\:\right)}}$	$\:\mu\:$	$\:\mu\:+\frac{\text{L}\text{o}\text{g}\left[\text{L}\text{o}\text{g}\left[2\right]\right]}{r}$		$\:t\in\:\left(-\infty\:,\infty\:\right)$	^32^
12	Half Cauchy	$\:\frac{2m\text{A}\text{r}\text{c}\text{T}\text{a}\text{n}\left[r\left(t-\mu\:\right)\right]}{\pi\:}$	$\:\frac{2mr}{\pi\:\left(1+{r}^{2}{\left(t-\mu\:\right)}^{2}\right)}$	$\:\mu\:$	$\:\mu\:+\frac{1}{r}$		$\:t\ge\:\mu\:$	^38^
13	Half logistic	$\:m\text{T}\text{a}\text{n}\text{h}\left[\frac{rt}{2}\right]$	$\:\frac{mr}{1+\text{C}\text{o}\text{s}\text{h}\left[rt\right]}$	$\:0$	$\:\frac{\text{L}\text{o}\text{g}\left[3\right]}{r}$		$\:t\ge\:0$	^39^
14	Inverse exponential	$\:m{e}^{-\frac{1}{rt}}$	$\:\frac{m{e}^{-\frac{1}{rt}}}{r{t}^{2}}$	$\:\frac{1}{2r}$	$\:\frac{1}{r\text{L}\text{o}\text{g}\left[2\right]}$		$\:t\ge\:0$	^40^
15	Inverse paralogistic	$\:m{\left(1+{\left(rt\right)}^{-a}\right)}^{-a}$	$\:\frac{m{a}^{2}{\left(rt\right)}^{{a}^{2}}}{{\left(1+{\left(rt\right)}^{a}\right)}^{1+a}t}$	$\:\frac{{\left(a-1\right)}^{1/a}}{r}$	$\:\frac{{\left({2}^{1/a}-1\right)}^{-1/a}}{r}$	$\:a>1$	$\:t\ge\:0$	^27^
16	Logistic	$\:\frac{m}{1+{e}^{-r\left(t-\mu\:\right)}}$	$\:\frac{mr}{2+2\text{C}\text{o}\text{s}\text{h}\left[r\left(t-\mu\:\right)\right]}$	$\:\mu\:$	$\:\mu\:$		$\:t\in\:\left(-\infty\:,\infty\:\right)$	^26^
17	Log-logistic	$\:m-\frac{m}{1+{\left(rt\right)}^{a}}$	$\:\frac{ma{r}^{a}{t}^{a-1}}{{\left(1+{\left(rt\right)}^{a}\right)}^{2}}$	$\:\frac{{\left(\frac{a-1}{a+1}\right)}^{1/a}}{r}$	$\:\frac{1}{r}$	$\:a>0$	$\:t\ge\:0$	^28^
18	Paralogistic	$\:m-m{\left(1+{\left(rt\right)}^{a}\right)}^{-a}$	$\:\frac{m{a}^{2}{\left(rt\right)}^{a}}{{\left(1+{\left(rt\right)}^{a}\right)}^{a+1}t}$	$\:\frac{{\left(\frac{a-1}{{a}^{2}+1}\right)}^{1/a}}{r}$	$\:\frac{{\left({2}^{1/a}-1\right)}^{1/a}}{r}$	$\:a>1$	$\:t\ge\:0$	^41^
19	Rayleigh 2p	$\:m\left(1-{e}^{-\frac{{\left(rt\right)}^{2}}{2}}\right)$	$\:m{r}^{2}t{e}^{-\frac{{\left(rt\right)}^{2}}{2}}$	$\:\frac{1}{r}$	$\:\frac{\sqrt{\text{L}\text{o}\text{g}\left[4\right]}}{r}$		$\:t\ge\:0$	^41^
20	Rayleigh 3p	$\:m\left(1-{e}^{-\frac{{r}^{2}{\left(t-\mu\:\right)}^{2}}{2}}\right)$	$\:m{r}^{2}\left(t-\mu\:\right){e}^{-\frac{{r}^{2}{\left(t-\mu\:\right)}^{2}}{2}}$	$\:\mu\:+\frac{1}{r}$	$\:\mu\:+\frac{\sqrt{\text{L}\text{o}\text{g}\left[4\right]}}{r}$		$\:t\ge\:\mu\:$	^41^
21	Weibull	$\:m\left(1-{e}^{-{\left(rt\right)}^{a}}\right)$	$\:ma{r}^{a}{t}^{a-1}{e}^{-{\left(rt\right)}^{a}}$	$\:\frac{{\left(\frac{a-1}{a}\right)}^{1/a}}{r}$	$\:\frac{{\text{L}\text{o}\text{g}\left[2\right]}^{1/a}}{r}$	$\:a>1$	$\:t\ge\:0$	^26^

^1^$\:t$ is time (h); $\:F\left(t\right)$ is the cumulative in vitro gas production (GP) function; $\:{F}^{{\prime\:}}\left(t\right)$ is rate of GP ($\:=\frac{dF\left(t\right)}{dt}$); $\:{t}^{*}$ denotes the inflection point, which is the time at which the rate of GP reaches its maximum, with the corresponding GP amount of $\:{GP}^{*}$; $\:{t}_{0.5}$ is the half-life, representing the time when half of the maximum GP ($\:{GP}_{0.5}=\frac{m}{2}$) is achieved. $\:m$ is the asymptotic value of GP as time progresses indefinitely (measured in mL); $\:r$ is rate constant (unit is 1/h); $\:\mu\:$ (measured in h) is referred to as lag time, in the Exponential 3p and Half Cauchy models, it represents lag time as a discrete value, indicating that a specific time period must elapse before GP begins, during which measurable gas is not yet present. In the Cauchy, Gumbel, and Logistic models, $\:\mu\:$ is treated as an estimated parameter corresponding to the inflection point ($\:{t}^{*}$), which can influence the shape and rate of the GP curve. In the Rayleigh 3p model, $\:\mu\:$ is also an estimated parameter but is not directly related to lag time or the inflection point; $\:a$ and $\:p$ are shape parameters which are dimensionless. All parameters are real numbers ($\:\in\:\mathbb{R}$) within the specified range.

^2^ In all models, $\:m,r>0$; parameter assumptions are derived from our current GP problem and may vary in different contexts. For example, in the literature on the Burr XII, Inverse paralogistic, Dagum, Paralogistic, Weibull models, the domain for model fitting is commonly considered to be $\:a>0$. However, to obtain meaningful values for $\:{t}^{*}$ and $\:{GP}^{*}$, it is essential that $\:a>1$, a common condition in GP curve fitting.

^3^ The models presented here have been adjusted in some cases to modify their parameters and structure, making them more comparable, while retaining the core concept of the original models.

#### Model fitting

We analyzed the 849 GP curves using 21 distinct mathematical models to evaluate their effectiveness in capturing the dynamics of GP across data samples. Each model was fitted to the data using MATLAB’s (MATLAB^®^ Version 23.2, R2023b, The MathWorks Inc.) ‘*fitnlm’* function, which facilitates nonlinear regression by optimizing model parameters to match best the observed data^18^. The ‘*fitnlm’* function employs a specified model formula and initial parameter guesses to minimize the residual sum of squares between observed and predicted values. We configured the optimization process using the *‘statset’* function, which allowed us to set parameters like maximum iterations and tolerance levels to ensure convergence and accuracy. To assess the performance of each model, we employed several statistical metrics of coefficient of determination (R²), root mean square error (RMSE), Log-likelihood, and Bayesian information criterion (BIC). Among these, BIC was particularly emphasized as the primary criterion for model selection because BIC accounts for both the goodness of fit (through error reduction) and model complexity (number of parameters), making it a robust metric for evaluating models across datasets with varying structures and parameter counts^19,20^.

The BIC was calculated for each model fit using the standard formulation:

$$\:BIC=k{\ln}\left(n\right)-2{\ln}\left(\widehat{L}\right)$$

where $\:k$ is the number of model parameters, $\:n$ is the number of data points per curve, and $\:\widehat{L}$ is the maximized value of the likelihood function under the assumption of normally distributed, independent residuals. The underlying error model assumes additive, homoscedastic, and normally distributed errors. While the time-series nature of GP data might suggest potential autocorrelation, each curve was fitted independently using standardized sampling intervals. Therefore, the assumption of residual independence across time points is commonly accepted and appropriate in this context. This formulation ensures that model comparisons via BIC are statistically sound and consistent with nonlinear regression practices in similar biological modeling studies. By balancing model accuracy and complexity, BIC helps confirm that the selected models are precise and parsimonious, avoiding overfitting while yielding reliable descriptions of the GP data.

### Model refinement using statistical and machine learning analyses

To refine model selection and enhance the interpretation of the GP data, we employed two distinct approaches: post hoc statistical analysis^21^ and machine learning decision tree analysis^22,23^. Both methods were used to identify which model (or group of models) provided the best fit across different feed categories, ultimately aiding in the decision-making process for accurate model selection.

#### Post hoc statistical analysis

We conducted a post hoc statistical analysis^21^ to investigate how BIC values varied with different models and feed categories, and to explore the interaction between these factors. The analysis involved a factorial model where the calculated BIC was modeled as a function of the main effects of model name (21 model names, Table 1), feed category (6 categories), and their interaction.

$$\:{Calculated\:BIC}_{ijk}=\:\mu\:+\:{\alpha\:}_{i}+\:{\beta\:}_{j}+{\left(\alpha\:\beta\:\right)}_{ij}+{\epsilon\:}_{ijk}\:$$

where $\:{Calculated\:BIC}_{ijk}$ is the calculated BIC value for the $\:{k}^{th}$ sample, $\:{i}^{th}\:$model, and $\:{j}^{th}$ feed category; $\:\mu\:$ is overall mean; $\:{\alpha\:}_{i}$ is the effect of the $\:{i}^{th}$ model; $\:{\beta\:}_{j}$ is the effect of the $\:{j}^{th}$ feed category; $\:{\left(\alpha\:\beta\:\right)}_{ij}$ is the interaction between model and feed category; and $\:{\epsilon\:}_{ijk}$ is the random error term assumed to be normally distributed with constant variance.

In the post hoc statistical model, “model name” refers to the 21 evaluated nonlinear models (Table 1), and “feed category” refers to the six feed types defined earlier. These factors were treated as fixed effects in a factorial analysis of variance to assess their individual and interactive effects on the calculated BIC obtained from the process of fitting the models to the data. To further analyze significant differences identified in the initial ANOVA, we applied Tukey’s honestly significant difference (HSD) test^21^. This test provided a structured comparison of the feed categories and how model performance varied across them.

#### Machine learning decision tree analysis

We also employed a machine learning decision tree model^22,23^ to assess the relationships between model name, feed categories (inputs), and calculated BIC (output) values. The decision tree was implemented as a regression model, with the calculated BIC obtained from the process of fitting the models to the data as the continuous response variable and both “model name” (21 levels) and “feed category” (6 levels) as categorical input variables. The dataset, which included calculated BIC values from fitting all models on all samples, was divided into training (70%) and validation (30%) sets. The decision tree was trained on the training set to learn the patterns and relationships, and its performance was evaluated on the validation set to ensure accuracy and generalizability^22,24^. To optimize the decision tree, parameters such as tree depth and minimum samples per leaf were adjusted based on performance metrics obtained from the validation set. This optimization aimed to enhance the model’s predictive power and prevent overfitting^24,25^ensuring that the decision tree could effectively identify the most relevant partition (i.e. model name and feed category) influencing BIC values. We used a single tree rather than an ensemble method to maintain transparency and enable direct extraction of decision rules for model selection across feed categories. The decision tree method was executed under a predictive modeling algorithm developed using JMP^®^ Software (JMP^®^ Pro, Version 17.2.0, SAS Institute Inc., Cary, NC, 1989–2023).

To evaluate whether model selection improves the accuracy and reliability of GP analyses, we calculated relative performance improvement (RPI) percentages. The RPI represents the percentage by which the top-performing models outperform lower-performing models, relative to the average BIC across all models within each feed category. It is calculated using the following formula:

$$\:\text{R}\text{P}\text{I}=\frac{Difference\:\left(Lower\:-\:Top\right)}{Average\:BIC\:\left(All\:models\right)}\times\:100$$

In this context $\:Difference\:\left(Lower\:-\:Top\right)$ represents the difference between the average BIC of the lower-performing models and the average BIC of the top-performing models, and $\:Average\:BIC\:\left(All\:models\right)$ is the average BIC calculated across all models in the respective feed category.

## Results

### Parameter estimates and performance across feed categories

Table 2 summarizes the estimated and calculated parameters derived from various models applied to the six feed categories, describing GP dynamics. The table is also complemented by goodness-of-fit metrics of R², RMSE, Log-likelihood, and BIC, which collectively characterize the models’ performance across varying feed types.

**Table 2:** Summary of model parameter estimates and performance metrics across models and feed categories.

Model name	Feed category	Estimated parameters^1^					Calculated parameters^2^				Goodness of fit^3^			
		$\:m$ (mL)	$\:r$ (1/h)	$\:\mu\:$ (h)	$\:a$	$\:p$	$\:{t}_{0.5}$ (h)	$\:{GP}_{0.5}$ (mL)	$\:{t}^{*}$ (h)	$\:{GP}^{*}$ (mL)	R^2^	RMSE (mL)	Log- likelihood	BIC
Burr III	Compound	77.1			0.953	6.135	9.32	38.54	2.55	9.58	0.996	1.69	− 15.4	37.4
	Corn	88.8			1.333	13.813	9.28	44.39	4.47	15.55	0.995	2.68	− 19.7	45.9
	Processed protein	64.6			0.779	5.097	12.21	32.28	1.87	5.67	0.997	1.15	− 11.7	29.9
	Legumes	78.0			1.172	6.074	6.08	39.02	2.29	12.17	0.998	1.41	− 12.7	32.0
	Soft cereal	83.6			1.315	7.862	6.02	41.79	2.78	14.52	0.994	2.69	− 19.5	45.7
	Others	74.5			0.960	5.644	8.29	37.24	2.23	9.34	0.996	1.87	− 15.7	37.9
Burr XII	Compound	70.4	0.109		1.463	1.485	8.25	35.20	2.70	11.36	0.999	1.04	− 10.1	29.0
	Corn	84.4	0.111		2.097	2.155	8.97	42.20	5.44	22.01	0.998	1.64	− 14.3	37.3
	Processed protein	57.1	0.083		1.183	2.226	10.15	28.54	1.41	4.65	0.999	0.74	− 7.0	22.7
	Legumes	76.8	0.241		1.970	0.788	6.02	38.42	2.56	15.12	0.999	1.14	− 9.6	27.9
	Soft cereal	83.1	0.255		2.459	0.592	6.03	41.55	3.22	18.56	0.996	2.48	− 17.9	44.7
	Others	71.9	0.193		1.604	0.967	8.07	35.96	2.51	11.61	0.998	1.48	− 13.2	35.2
Cauchy	Compound	65.9	0.279	7.926			7.93	32.97	7.93	32.97	0.969	5.06	− 25.5	57.6
	Corn	81.3	0.338	8.869			8.87	40.66	8.87	40.66	0.980	5.35	− 26.0	58.5
	Processed protein	50.5	0.232	8.404			8.40	25.25	8.40	25.25	0.961	4.25	− 23.8	54.1
	Legumes	71.5	0.392	5.939			5.94	35.74	5.94	35.74	0.969	5.40	− 26.1	58.7
	Soft cereal	77.4	0.459	5.954			5.95	38.68	5.95	38.68	0.974	5.56	− 26.3	59.2
	Others	64.3	0.306	7.099			7.10	32.15	7.10	32.15	0.967	4.95	− 25.3	57.2
Dagum	Compound	70.2	0.108		1.703	0.834	8.24	35.08	2.67	11.36	0.999	1.00	− 9.7	28.2
	Corn	83.7	0.105		2.409	0.867	8.95	41.87	5.75	23.64	0.998	1.59	− 13.9	36.6
	Processed protein	56.7	0.157		1.504	0.993	9.79	28.35	1.14	3.75	0.999	0.71	− 6.5	21.8
	Legumes	75.9	0.644		1.553	3.243	5.94	37.95	2.46	14.43	0.999	1.20	− 10.0	28.8
	Soft cereal	81.6	0.637		1.740	4.473	5.95	40.79	3.12	17.86	0.995	2.62	− 18.5	45.7
	Others	70.2	0.311		1.535	1.776	7.74	35.08	2.50	11.68	0.998	1.45	− 13.2	35.2
Exponential 2p	Compound	68.2	0.085				8.21	34.09	0.00	0.00	0.995	1.85	− 16.8	37.9
	Corn	86.0	0.071				9.93	43.01	0.00	0.00	0.977	5.36	− 26.7	57.8
	Processed protein	51.8	0.082				8.55	25.91	0.00	0.00	0.996	1.14	− 12.3	29.0
	Legumes	73.5	0.121				5.85	36.76	0.00	0.00	0.988	3.06	− 20.6	45.6
	Soft cereal	80.6	0.116				6.05	40.31	0.00	0.00	0.978	4.80	− 25.5	55.5
	Others	66.3	0.096				7.26	33.17	0.00	0.00	0.992	2.36	− 19.0	42.5
Exponential 3p	Compound	67.9	0.089	0.244			8.12	33.97	0.24	0.00	0.996	1.83	− 16.0	38.6
	Corn	85.0	0.080	0.817			9.57	42.52	0.82	0.00	0.983	4.90	− 25.2	57.0
	Processed protein	51.9	0.081	− 0.042			8.57	25.93	− 0.04	0.00	0.997	1.16	− 11.9	30.3
	Legumes	73.3	0.127	0.253			5.82	36.65	0.25	0.00	0.990	3.05	− 20.0	46.5
	Soft cereal	80.2	0.125	0.401			6.00	40.11	0.40	0.00	0.981	4.78	− 24.8	56.2
	Others	66.2	0.099	0.164			7.21	33.09	0.16	0.00	0.992	2.42	− 18.7	43.9
Exponentiated exponential	Compound	67.2	0.106		1.233		7.97	33.59	1.83	7.83	0.997	1.45	− 14.0	34.6
	Corn	81.9	0.148		2.176		8.87	40.95	5.24	21.25	0.997	2.10	− 17.4	41.3
	Processed protein	52.2	0.078		0.971		8.66	26.10	0.49	1.58	0.998	0.93	− 10.1	26.8
	Legumes	72.2	0.174		1.522		5.77	36.12	1.91	11.79	0.995	2.15	− 17.4	41.5
	Soft cereal	78.2	0.205		1.942		5.85	39.11	3.06	18.35	0.990	3.42	− 21.8	50.2
	Others	65.7	0.113		1.189		7.16	32.85	1.39	6.84	0.994	2.02	− 17.2	41.0
Frechet	Compound	79.2	0.161		0.850		9.76	39.58	2.48	8.92	0.996	1.90	− 16.5	39.6
	Corn	89.1	0.145		1.294		9.29	44.54	4.44	15.04	0.994	2.81	− 20.1	46.8
	Processed protein	68.1	0.133		0.666		13.96	34.03	1.86	5.37	0.996	1.29	− 12.7	31.9
	Legumes	79.2	0.237		1.067		6.19	39.58	2.23	11.30	0.997	1.57	− 13.9	34.5
	Soft cereal	84.4	0.227		1.226		6.06	42.18	2.71	13.58	0.993	2.81	− 19.9	46.5
	Others	76.5	0.182		0.853		8.70	38.23	2.19	8.71	0.995	2.02	− 16.3	39.1
Generalized logistic	Compound	68.3			0.084		8.32	34.16	2.48	13.17	0.991	2.47	− 19.6	43.5
	Corn	86.2			0.070		10.03	43.08	2.66	14.93	0.974	5.70	− 27.3	58.9
	Processed protein	51.9			0.081		8.66	25.96	2.52	9.74	0.993	1.58	− 15.6	35.6
	Legumes	73.7			0.118		5.98	36.87	2.14	17.23	0.981	4.02	− 23.6	51.6
	Soft cereal	80.8			0.114		6.18	40.42	2.18	18.42	0.971	5.54	− 26.9	58.2
	Others	66.5			0.095		7.37	33.24	2.36	13.71	0.987	2.93	− 21.1	46.6
Gompertz	Compound	67.6	0.083		1.011		8.14	33.78	0.12	0.53	0.996	1.81	− 15.9	38.4
	Corn	80.4	0.042		1.139		9.07	40.19	7.24	32.87	0.992	3.28	− 21.4	49.3
	Processed protein	54.3	0.081		0.989		9.15	27.13	0.00	0.00	0.997	1.02	− 11.0	28.6
	Legumes	72.8	0.106		1.048		5.92	36.42	0.95	6.57	0.992	2.72	− 19.1	44.8
	Soft cereal	77.9	0.088		1.105		5.99	38.97	2.54	14.26	0.984	4.39	− 24.1	54.8
	Others	67.9	0.091		1.006		7.63	33.96	0.61	2.87	0.994	2.15	− 17.5	41.5
Gumbel	Compound	63.9	0.211	9.925			8.18	31.97	9.92	40.42	0.967	5.26	− 25.8	58.3
	Corn	79.2	0.247	10.594			9.11	39.62	10.59	50.09	0.983	4.95	− 25.2	57.0
	Processed protein	48.3	0.192	10.317			8.40	24.16	10.32	30.55	0.957	4.46	− 24.2	55.0
	Legumes	69.1	0.293	7.463			6.14	34.56	32.14	54.07	0.959	6.22	− 27.3	61.2
	Soft cereal	74.6	0.348	7.126			6.04	37.29	53.46	66.59	0.962	6.74	− 28.0	62.7
	Others	62.6	0.220	9.114			7.44	31.28	9.11	39.55	0.959	5.47	− 26.2	59.0
Half Cauchy	Compound	75.2	0.114	0.283			9.21	37.61	0.28	0.00	0.997	1.60	− 14.6	35.7
	Corn	95.0	0.101	0.914			10.96	47.49	0.91	0.00	0.985	4.69	− 24.8	56.2
	Processed protein	57.6	0.103	− 0.026			9.75	28.80	− 0.03	0.00	0.998	0.94	− 10.0	26.6
	Legumes	79.7	0.169	0.338			6.40	39.87	0.34	0.00	0.992	2.70	− 18.1	42.9
	Soft cereal	87.5	0.166	0.509			6.63	43.73	0.51	0.00	0.984	4.39	− 24.0	54.6
	Others	72.8	0.129	0.221			8.08	36.40	0.22	0.00	0.995	1.99	− 16.3	39.1
Half logistic	Compound	66.4	0.140				7.96	33.22	0.00	0.00	0.995	1.82	− 16.8	38.0
	Corn	83.4	0.118				9.44	41.70	0.00	0.00	0.987	3.98	− 24.0	52.4
	Processed protein	50.4	0.134				8.27	25.20	0.00	0.00	0.990	1.81	− 16.3	37.1
	Legumes	72.1	0.193				5.80	36.03	0.00	0.00	0.990	2.83	− 20.6	45.6
	Soft cereal	78.9	0.187				5.95	39.45	0.00	0.00	0.984	4.12	− 24.2	52.7
	Others	64.7	0.157				7.07	32.37	0.00	0.00	0.990	2.42	− 19.5	43.4
Inverse exponential	Compound	74.3	0.173				8.47	37.17	2.94	10.06	0.993	2.14	− 18.4	41.1
	Corn	95.9	0.137				10.68	47.97	3.70	12.99	0.991	3.27	− 22.2	48.7
	Processed protein	55.9	0.172				8.51	27.95	2.95	7.57	0.986	2.32	− 18.9	42.2
	Legumes	79.9	0.239				6.15	39.94	2.13	10.81	0.995	1.99	− 16.5	37.4
	Soft cereal	88.3	0.224				6.54	44.15	2.27	11.95	0.990	3.13	− 21.5	47.3
	Others	72.1	0.195				7.49	36.06	2.60	9.76	0.992	2.34	− 19.0	42.4
Inverse paralogistic	Compound	72.4	0.158		1.333		8.51	36.19	2.75	11.26	0.998	1.15	− 11.9	30.3
	Corn	85.9	0.168		1.726		9.05	42.97	4.96	19.19	0.997	1.94	− 16.7	39.9
	Processed protein	58.2	0.121		1.163		9.97	29.11	1.70	5.41	0.998	0.82	− 8.8	24.2
	Legumes	75.8	0.244		1.495		5.91	37.88	2.43	14.10	0.998	1.20	− 11.2	29.0
	Soft cereal	81.5	0.252		1.662		5.93	40.75	3.06	17.43	0.995	2.51	− 18.9	44.4
	Others	70.5	0.174		1.318		7.63	35.23	2.31	10.61	0.997	1.57	− 14.5	35.7
Logistic	Compound	64.3	0.289	7.879			7.88	32.14	7.88	32.14	0.978	4.31	− 24.0	54.7
	Corn	79.6	0.328	8.834			8.83	39.80	8.83	39.80	0.989	3.91	− 23.1	52.7
	Processed protein	48.9	0.248	8.286			8.29	24.47	8.29	24.47	0.968	3.79	− 22.7	52.0
	Legumes	70.0	0.399	5.934			5.93	34.98	5.93	34.98	0.975	4.85	− 25.1	56.7
	Soft cereal	75.9	0.445	5.957			5.96	37.96	5.96	37.96	0.977	5.31	− 25.9	58.3
	Others	62.8	0.314	7.089			7.09	31.41	7.09	31.41	0.972	4.43	− 24.3	55.2
Log− logistic	Compound	71.1	0.121		1.500		8.35	35.57	2.81	11.73	0.999	1.03	− 10.9	28.4
	Corn	84.3	0.113		2.094		8.95	42.15	5.40	21.91	0.998	1.63	− 15.1	36.7
	Processed protein	57.5	0.104		1.251		9.81	28.75	1.67	5.38	0.999	0.76	− 8.1	22.8
	Legumes	74.9	0.174		1.707		5.89	37.46	2.50	15.07	0.998	1.24	− 11.8	30.2
	Soft cereal	80.5	0.171		1.954		5.91	40.23	3.25	19.35	0.994	2.56	− 19.1	44.8
	Others	69.5	0.134		1.472		7.53	34.74	2.33	10.97	0.997	1.49	− 14.2	35.1
Paralogistic	Compound	69.8	0.090		1.400		8.20	34.92	2.68	11.30	0.999	1.03	− 10.9	28.3
	Corn	82.8	0.074		1.841		8.91	41.41	5.51	22.72	0.998	1.76	− 15.7	38.0
	Processed protein	56.7	0.087		1.209		9.65	28.37	1.57	5.09	0.999	0.73	− 7.7	22.1
	Legumes	74.0	0.123		1.531		5.86	37.02	2.39	14.67	0.998	1.48	− 13.3	33.2
	Soft cereal	79.5	0.115		1.701		5.91	39.77	3.16	19.23	0.993	2.89	− 20.2	47.0
	Others	68.5	0.102		1.369		7.44	34.24	2.18	10.46	0.997	1.51	− 14.4	35.3
Rayleigh 2p	Compound	63.2	0.160				7.42	31.62	6.30	24.88	0.963	5.11	− 26.2	56.9
	Corn	79.5	0.138				8.59	39.76	7.30	31.29	0.990	3.41	− 22.5	49.4
	Processed protein	47.6	0.157				7.56	23.80	6.42	18.73	0.939	4.88	− 25.7	55.7
	Legumes	69.0	0.210				5.69	34.49	4.83	27.14	0.965	5.13	− 26.1	56.7
	Soft cereal	75.6	0.204				5.82	37.80	4.94	29.75	0.976	4.96	− 25.9	56.2
	Others	61.7	0.177				6.72	30.87	5.70	24.30	0.956	5.16	− 26.3	57.1
Rayleigh 3p	Compound	64.8	0.109	− 3.120			7.83	32.39	6.18	25.49	0.986	3.34	− 21.7	50.1
	Corn	80.2	0.118	− 1.347			8.82	40.12	7.29	31.57	0.994	2.92	− 20.3	47.1
	Processed protein	49.5	0.093	− 4.502			8.34	24.74	6.40	19.47	0.978	3.10	− 20.8	48.3
	Legumes	70.5	0.152	− 2.113			5.86	35.27	4.66	27.76	0.984	3.81	− 22.9	52.3
	Soft cereal	76.8	0.163	− 1.391			5.93	38.38	4.83	30.21	0.984	4.43	− 24.2	55.0
	Others	63.3	0.120	− 2.885			7.01	31.63	5.52	24.89	0.981	3.58	− 22.3	51.3
Weibull	Compound	67.1	0.091		1.114		8.00	33.56	1.44	6.30	0.997	1.56	− 14.6	35.9
	Corn	81.3	0.090		1.554		8.92	40.63	5.72	24.06	0.996	2.48	− 18.8	44.2
	Processed protein	52.4	0.079		0.967		8.72	26.21	0.31	1.03	0.998	0.91	− 9.9	26.3
	Legumes	72.2	0.129		1.226		5.81	36.09	1.72	11.08	0.994	2.35	− 18.1	42.9
	Soft cereal	77.9	0.132		1.414		5.89	38.97	3.04	18.91	0.988	3.74	− 22.6	51.9
	Others	65.8	0.099		1.079		7.20	32.88	1.19	6.01	0.994	2.06	− 17.3	41.3

^1^$\:m$ is the asymptotic value of in vitro gas production (GP) as time progresses indefinitely; $\:r$ is rate constant; $\:\mu\:$ represents discrete lag time in the Exponential 3p and Half Cauchy models, indicating a delay period before GP begins, notably, the estimated $\:\mu\:$ for these models was not significantly different from zero in our study. $\:\mu\:$ in other models Cauchy, Gumbel, Logistic, Rayleigh 3p is an estimated parameter (see Table 1 footnote in main article for more definition); $\:a$ and $\:p$ are shape parameters which are dimensionless.

^2^$\:{t}_{0.5}$ is the half-life, representing the time when half of the maximum GP ($\:{GP}_{0.5}=\frac{m}{2}$) is achieved; $\:{t}^{*}$ denotes the inflection point, which is the time at which the rate of GP reaches its maximum, with the corresponding GP amount denoted as $\:{GP}^{*}$.

^3^ R²: Coefficient of determination; RMSE: Root mean squared error; BIC: Bayesian information criterion.

#### The goodness-of-fit metrics

The values indicate that all models exhibit relatively successful fits across the different feed categories. With an average R² of 0.988, ranging from 0.939 to 0.999, it is evident that all models effectively account for a high proportion of variance in the GP data. The low RMSE, averaging at 2.83 mL (with a range of 0.71 to 6.74 mL), signifies low prediction errors, reinforcing the models’ reliability. Moreover, the Log-likelihood values, which averaged − 18.6 and ranged from − 28.0 to − 6.5, indicate good model fits, even with the varying complexities of GP curves. The BIC values, averaging 43.5 with a range of 21.8 to 62.7, highlight a balance between model accuracy and simplicity.

### Interactions between model and feed categories

Post hoc statistical analysis revealed that the obtained BIC values varied significantly (*P* < 0.01) depending on the model and feed category (Table 3). Additionally, a significant interaction (*P* < 0.01) between model type and feed category was observed, indicating that the accuracy of GP predictions regarding BIC is complex and interactively influenced by both the model type and the feed type under investigation. Variations in feed composition, fermentation characteristics, model structure, and the fundamental assumptions of each model contribute to the observed differences in BIC values.

**Table 3:** Summary of analysis of variance for bayesian information criterion (BIC) values across feed categories and model names.

Source of variation	DF	*P* value
Feed category	5	< 0.01
Model name	20	< 0.01
Feed category × Model name	100	< 0.01

#### Rate constant ($\:\varvec{r}$)

The Soft cereal and Legumes categories exhibited the highest mean $\:r$ values, averaging 0.238 h⁻¹ and 0.230 h⁻¹, respectively. Conversely, feed categories such as Processed protein (0.127 h⁻¹), Compound (0.141 h⁻¹), and Corn (0.141 h⁻¹) showed the lowest values. It is important to note that the parameter denoted as $\:r$ does not serve an identical mathematical role across all models; therefore, comparisons of $\:r$ values between different models should be interpreted with caution. However, comparisons of $\:r$ values within the same model across different feeds may be considered meaningful.

#### Lag time ($\:\varvec{\mu\:}$)

The $\:\mu\:$ (measured in h) is defined as the period that must elapse before GP begins. In the Exponential 3p and Half Cauchy models, $\:\mu\:$ represents a lag time as a discrete value, indicating a specific time during which measurable gas is not yet present. The estimated $\:\mu\:$ across these models shows small variations in initiation periods before GP starts, ranging from 0.0 to 0.9 h across different feed types. Notably, the estimated $\:\mu\:$ for these models was not significantly different from zero in our study, suggesting that the observed delays in GP may not be practically relevant and could indicate a rapid onset of fermentation across the samples tested. In the Cauchy, Gumbel, and Logistic models, µ is treated as an estimated parameter corresponding to the inflection point ($\:{t}^{*}$), which can influence the shape and rate of the GP curve. It can be discussed in the context of how quickly and when the rate of GP reaches its maximum. In the Rayleigh 3p model, µ is also an estimated parameter but is not directly related to lag time or the inflection point.

#### Shape parameters ($\:\varvec{a}$ and $\:\varvec{p}$)

The shape parameter ($\:a$) values indicate the steepness of the GP curve, with Corn exhibiting the highest mean value of 1.62, indicating rapid initial GP. This is followed by Soft cereal (1.52), Legumes (1.31), and Compound (1.15), whereas the Processed protein category displayed the lowest mean a value of 0.97, indicating a slower initial GP rate. These differences emphasize the fermentation characteristics associated with each feed type, with higher $\:a$ value correlating with more favorable fermentation dynamics. The shape parameter ($\:p$) values, averaging 4.11 across feed categories, did not reveal significant differences between feed types, suggesting a consistent influence of $\:p$ on GP across various feeds. However, disregarding the feed effect, the models Burr II (8.08), Dagum (3.26), and Burr XII (0.99) yielded different $\:p$ values (*P* < 0.05).

### Post hoc model ranking

**Fig. 4:**
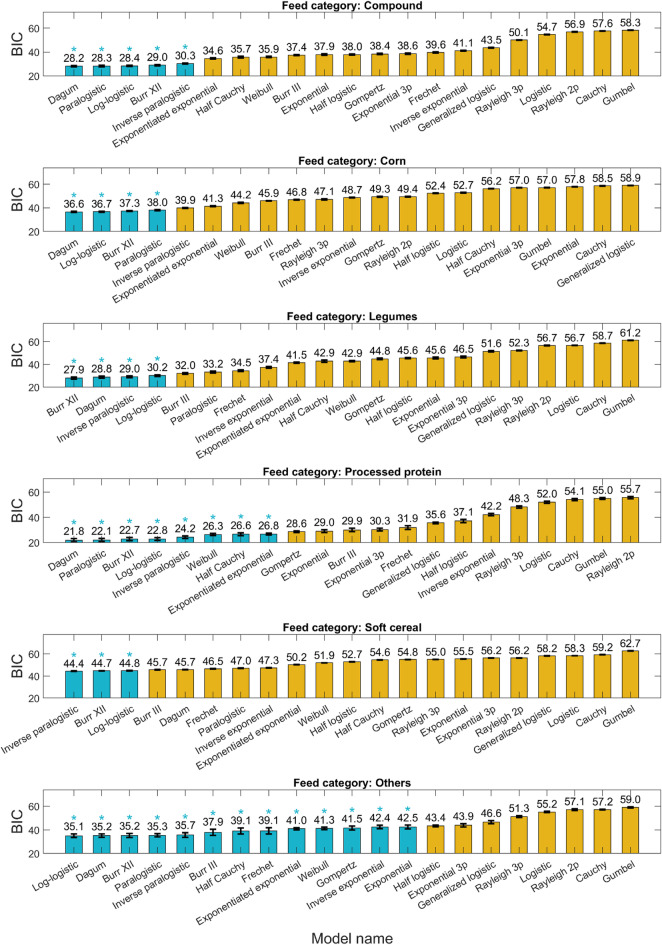
Bayesian information criterion (BIC) values for different models and feed categories. Models marked with an asterisk (*) indicate groups of models with statistically similar performance (*P* < 0.05) based on Tukey’s Honestly Significant Difference (HSD) test.

The Post Hoc statistical analysis revealed some findings regarding how different models performed across various feed categories, with their effectiveness assessed through BIC values. These results, summarized in Fig. 4, show the variability in model performance across feed categories, providing an understanding of the interaction between models and feed categories.

In the Compound feed category, five models were identified as the best fits, achieving an average BIC of 28.8. This group comprised the Dagum, Paralogistic, Log-logistic, Burr XII, and Inverse paralogistic models, indicating a robust fitting capability. Four models emerged as the best fits for the Corn feed category, demonstrating a higher average BIC of 37.2. This group included the Dagum, Log-logistic, Burr XII, and Paralogistic models, suggesting that while effective, these models exhibited slightly less fitting performance compared to those in the Compound category. The Processed protein category displayed a diverse fitting capability, with eight models exhibiting effective performance and achieving an average BIC of 24.2. The models in this category included the Dagum, Paralogistic, Burr XII, Log-logistic, Inverse paralogistic, Weibull, and Half Cauchy, highlighting the range of suitable models for this feed type. In the Legume category, four models were recognized for their fitting efficacy, resulting in an average BIC of 29.0. Notably, the Burr XII model exhibited the best performance, closely followed by the Dagum, Inverse paralogistic, and Log-logistic models. For the Soft cereal category, three models distinguished themselves as top performers, yielding a considerably higher average BIC of 44.6. The leading model, Inverse paralogistic, was followed closely by the Burr XII and Log-logistic models, indicating a variation in fitting capability relative to other feed categories. For the Others feed category, a wider range of models showed comparable performance (by an average BIC of 38.6). Among them, Log-logistic, Dagum, Burr XII, and Paralogistic were frequently observed, similar to the previously mentioned feed categories.

### Machine learning decision tree analysis

The decision tree model identified the correlation between the performance of the 21 tested models across the six feed categories, as indicated by metrics obtained from the training (R² = 0.76; RMSE = 5.2) and validation (R² = 0.76; RMSE = 5.1) processes. The decision tree model’s structure included 54 splits in the training data, indicating a relatively complex model that partitioned the data to capture patterns and relationships between the input features (model name and feed category) and the output (BIC values)^24^. While a higher number of splits can enhance accuracy by identifying more decision points, it also poses a risk of overfitting^25^. However, the consistent R² and RMSE values between the training and validation sets suggest that the model’s complexity was well-balanced and did not compromise generalizability. Thus, the 54 splits demonstrated that the model successfully captured key patterns while maintaining its effectiveness.

Further analysis revealed the contributions of model name and feed category to BIC, assessed by their respective sum of squares and the number of splits. The model name emerged as the dominant contributor, accounting for 679,218 of the total sum of squares across 34 splits, representing approximately 65.2% of overall model performance. In contrast, the feed category contributed 363,254 to the total sum of squares across 20 splits, accounting for 34.8% of overall model performance.

**Fig. 5:**
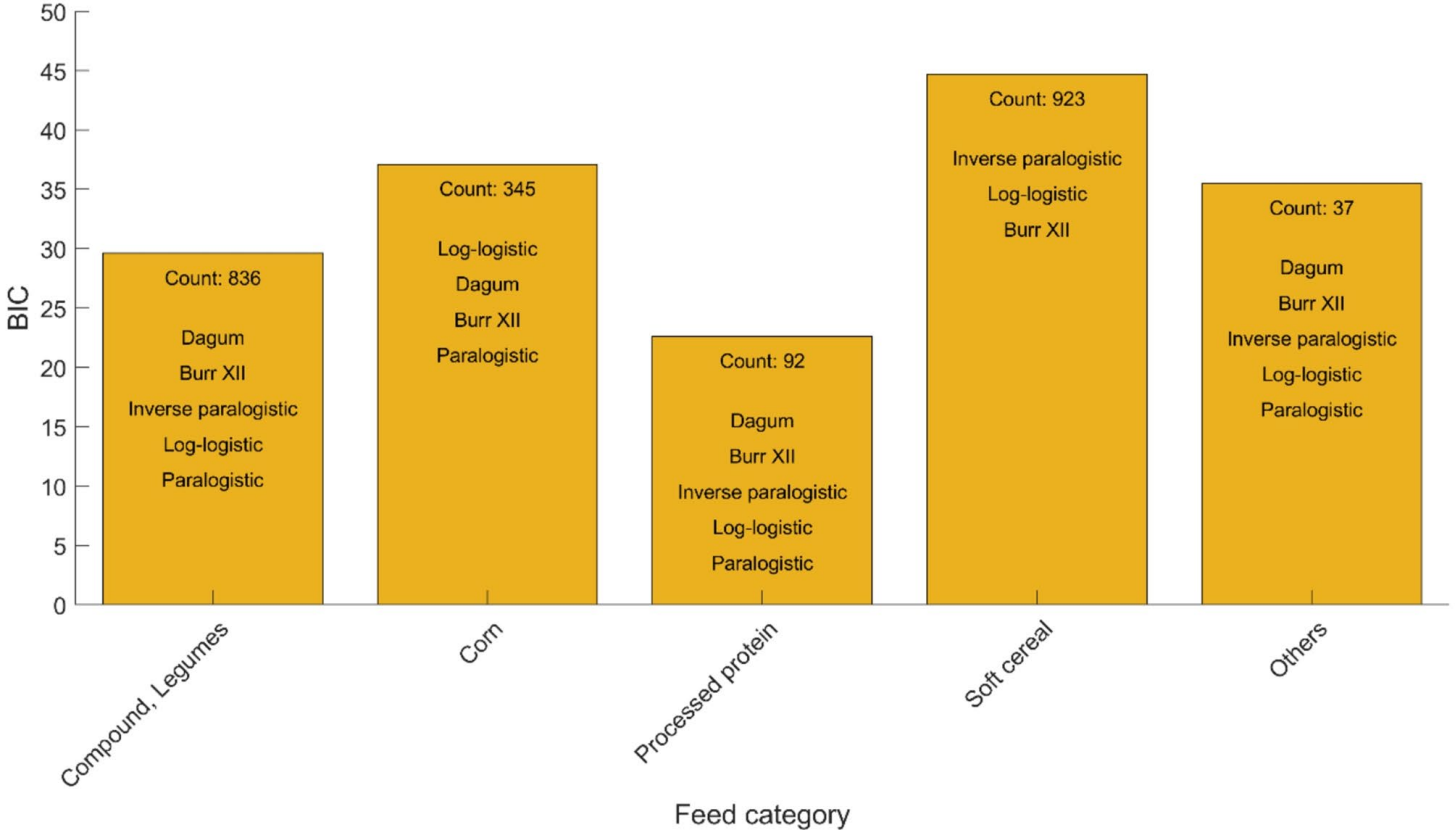
Top performing models by Bayesian information criterion (BIC) across different feed categories obtained from decision tree model analysis.

The analysis also generated additional detailed results on model selection across different feed categories. Due to the length and complexity of the decision tree, we focus solely on the end leaf reports to highlight the model or groups of models with the lowest BIC values in describing the GP curve. Key findings from the final leaf report are summarized in Tables 4 and 5 and illustrated in Fig. 5. In the Compound and Legumes categories, the top-performing models Dagum, Burr XII, Inverse paralogistic, Log-logistic, and Paralogistic resulted in an average BIC of 29.6 across 836 observations. In the Processed protein category, the same set of models (Dagum, Burr XII, Inverse paralogistic, Log-logistic, and Paralogistic) appeared as the top performers, with an average BIC of 22.6 based on 92 observations. For the Corn category, the models Log-logistic, Dagum, Burr XII, and Paralogistic showed the best performance, achieving an average BIC of 37.1 from 345 observations. For the Soft cereal category, the top-performing models were Inverse paralogistic, Log-logistic, and Burr XII, with an average BIC of 44.7 from 923 observations. Alternative models, including Burr III, Dagum, and Paralogistic showed relatively similar performance, with an average BIC of 46.1. In the Others category, the models Dagum, Burr XII, Inverse paralogistic, Log-logistic, and Paralogistic (similar to those in the Compound, Legumes, and Processed protein categories) produced an average BIC of 35.5 from 37 observations. The Inverse exponential model also showed comparable performance with an average BIC of 37.4.

**Table 4:** Decision tree leaf report of average bayesian information criterion (BIC) values and sample counts by feed category and model combination.

Leaf Label	Average BIC	Count	Note
Model name (Dagum, Burr XII, Inverse paralogistic, Log-logistic, Paralogistic) | Feed category (Processed protein)	22.6	92	Top performing
Model name (Weibull, Exponentiated exponential, Frechet, Burr III) | Feed category (Processed protein)	27.6	67	
Model name (Half Cauchy, Exponential, Gompertz, Exponential 3p) | Feed category (Processed protein)	28.8	81	
Model name (Dagum, Burr XII, Inverse paralogistic, Log-logistic, Paralogistic) | Feed category (Compound, Legumes)	29.6	836	Top performing
Model name (Burr III, Frechet) | Feed category (Legumes)	33.5	208	
Model name (Exponentiated exponential, Weibull) | Feed category (Compound)	35.2	118	
Model name (Dagum, Burr XII, Inverse paralogistic, Log-logistic, Paralogistic) | Feed category (Others)	35.5	37	Top performing
Model name (Generalized logistic, Half logistic) | Feed category (Processed protein)	36.9	29	
Model name (Log-logistic, Dagum, Burr XII, Paralogistic) | Feed category (Corn)	37.1	345	Top performing
Model name (Inverse exponential) | Feed category (Legumes, Others)	37.4	107	
Model name (Half Cauchy, Exponential, Half logistic, Gompertz, Exponential 3p) | Feed category (Compound)	37.6	294	
Model name (Burr III, Frechet) | Feed category (Others, Compound)	38.2	128	
Model name (Inverse paralogistic) | Feed category (Corn)	40.1	84	
Model name (Inverse exponential) | Feed category (Compound)	41.3	67	
Model name (Exponentiated exponential) | Feed category (Corn)	41.4	95	
Model name (Exponentiated exponential, Weibull) | Feed category (Legumes, Others)	42.2	225	
Model name (Inverse exponential) | Feed category (Processed protein)	42.5	11	
Model name (Generalized logistic) | Feed category (Compound)	43.9	64	
Model name (Weibull) | Feed category (Corn)	44.5	76	
Model name (Inverse paralogistic, Log-logistic, Burr XII) | Feed category (Soft cereal)	44.7	923	Top performing
Model name (Half Cauchy, Gompertz, Exponential, Half logistic, Exponential 3p) | Feed category (Others, Legumes)	44.8	578	
Model name (Burr III) | Feed category (Corn)	45.9	88	
Model name (Burr III, Dagum, Paralogistic) | Feed category (Soft cereal)	46.1	973	
Model name (Rayleigh 3p) | Feed category (Corn)	46.7	74	
Model name (Frechet, Inverse exponential) | Feed category (Soft cereal)	46.8	639	
Model name (Frechet) | Feed category (Corn)	46.9	83	
Model name (Inverse exponential) | Feed category (Corn)	48.4	84	
Model name (Gompertz) | Feed category (Corn)	49.1	77	
Model name (Rayleigh 3p) | Feed category (Processed protein, Compound)	49.8	78	
Model name (Rayleigh 2p) | Feed category (Corn)	49.9	73	
Model name (Exponentiated exponential) | Feed category (Soft cereal)	50.2	326	
Model name (Generalized logistic) | Feed category (Others, Legumes)	51.5	114	
Model name (Weibull) | Feed category (Soft cereal)	51.8	324	
Model name (Half logistic) | Feed category (Corn)	52.2	79	
Model name (Rayleigh 3p) | Feed category (Others, Legumes)	52.3	111	
Model name (Half logistic) | Feed category (Soft cereal)	52.6	307	
Model name (Logistic) | Feed category (Corn)	52.9	73	
Model name (Logistic, Rayleigh 2p) | Feed category (Processed protein)	54.1	33	
Model name (Half Cauchy) | Feed category (Soft cereal)	54.6	322	
Model name (Cauchy, Gumbel) | Feed category (Processed protein)	55.0	38	Lower performing
Model name (Gompertz, Rayleigh 3p) | Feed category (Soft cereal)	55.0	634	
Model name (Exponential, Exponential 3p) | Feed category (Soft cereal)	55.8	665	
Model name (Rayleigh 2p) | Feed category (Soft cereal)	56.2	337	
Model name (Logistic, Rayleigh 2p) | Feed category (Others, Compound, Legumes)	56.3	330	
Model name (Half Cauchy) | Feed category (Corn)	56.3	87	
Model name (Exponential 3p) | Feed category (Corn)	56.9	82	
Model name (Gumbel) | Feed category (Corn)	57.0	83	
Model name (Exponential) | Feed category (Corn)	57.9	81	
Model name (Cauchy, Gumbel) | Feed category (Compound, Others)	58.0	133	Lower performing
Model name (Logistic, Generalized logistic) | Feed category (Soft cereal)	58.3	635	
Model name (Cauchy) | Feed category (Legumes)	58.8	98	
Model name (Cauchy, Generalized logistic) | Feed category (Corn)	58.9	164	Lower performing
Model name (Cauchy) | Feed category (Soft cereal)	59.3	309	
Model name (Gumbel) | Feed category (Legumes)	61.0	100	Lower performing
Model name (Gumbel) | Feed category (Soft cereal)	62.6	325	Lower performing

**Table 5:** Summary of model performance across feed categories as partitioned by machine learning decision tree analysis^1^.

Feed category	Top performing models	Lower performing model(s)	Average BIC (Top models)	Average BIC (Lower models)	Average BIC (All models)	Difference (Lower – Top)	RPI, %
Compound	Dagum, Burr XII, Inverse paralogistic, Log-logistic, Paralogistic	Cauchy, Gumbel	29.6	58.0	40.1	28.4	70.8
Corn	Log-logistic, Dagum, Burr XII, Paralogistic	Cauchy, Generalized logistic	37.1	58.9	48.2	21.8	45.2
Processed protein	Dagum, Burr XII, Inverse paralogistic, Log-logistic, Paralogistic	Cauchy, Gumbel	22.6	55.0	34.4	32.4	94.2
Legumes	Dagum, Burr XII, Inverse paralogistic, Log-logistic, Paralogistic	Gumbel	29.6	61.0	42.9	31.4	73.2
Soft cereal	Inverse paralogistic, Log-logistic, Burr XII	Gumbel	44.7	62.6	52.0	17.9	34.4
Others	Dagum, Burr XII, Inverse paralogistic, Log-logistic, Paralogistic	Cauchy, Gumbel	35.5	58.0	43.6	22.5	51.6

^1^ BIC denotes Bayesian information criterion; RPI stands for relative performance improvement refers to the percentage by which the top-performing models outperform the lower-performing models relative to the average BIC across all models in each feed category. It is calculated as the difference between the average BIC of the lower and top-performing models, divided by the overall average BIC, and expressed as a percentage: $\:\left(\frac{Difference\:\left(Lower\:-\:Top\right)}{Average\:BIC\:\left(All\:models\right)}\times\:100\right)$.

### Selecting top-performing models

Based on the findings from both the post hoc statistical analysis and the machine learning decision tree analysis, the three top-performing models identified were Burr XII^26^Inverse paralogistic^27^and Log-logistic^28^. These models consistently emerged as optimal choices across various feed categories, showcasing their robustness in capturing the dynamics of GP effectively. Their consistent performance is supported by low BIC values, indicating their suitability for accurately modeling the GP curves in the studied feeds. The calculated RPI percentages presented in Table 5 demonstrate that using the three top models may lead to relative improvements. This is particularly evident in feed categories with higher RPI values. For instance, the Processed Protein category exhibited a remarkable 94.2% improvement when using top-performing models compared to lower-performing ones. Similarly, the Legumes and Compound categories showed substantial improvements of 73.2% and 70.8%, respectively.

## Discussion

### Importance of model selection despite universal fit

All candidate models in this study successfully fitted the GP data across all feed types, demonstrating their general applicability to in vitro fermentation kinetics. However, the substantial differences in RPI percentages presented in Table 5 underscore the importance of model selection in maximizing the accuracy and reliability of GP analyses. These significant performance gaps highlight that while all models may provide a reasonable fit, selecting the most appropriate model can markedly increase the precision of GP curve characterization. Additionally, the decision tree analysis revealed that model type substantially influenced performance more than feed category (65.2% vs. 34.8%). Several of the models assessed in this study, including the Cauchy, Exponential, Logistic, Log-logistic, Gompertz, and Weibull, have been applied in previous studies to describe GP kinetics due to their sigmoidal shape and biological interpretability^4–6,29^. However, our systematic evaluation revealed that these commonly used models were outperformed by less conventional alternatives such as the Burr XII and Inverse Paralogistic models, which have rarely been applied in the GP context. This finding underscores the importance of choosing the appropriate mathematical model in GP analysis, suggesting that model selection can significantly affect the accuracy of GP curve fitting, even more than the inherent differences among feed types.

### Highlights of the three selected models

In the context of GP models, flexibility refers to the model’s ability to effectively fit a wide range of data patterns and adjust the inflection point. Flexibility here implies that the model can allow $\:{GP}^{*}$ to occur at any proportion of the maximum GP ($\:m$). This adaptability is critical in capturing both the timing (when) and the proportion (where) of the GP inflection, ensuring that the model can represent diverse behavior across different feed types or conditions accurately. The three models that emerged beneficial in the present study —Burr XII, Inverse paralogistic, and Log-logistic—are considered flexible, however, the Burr XII model is regarded more flexible due to its two shape parameters, $\:a$ and $\:p$, which offer greater control over the location and nature of the inflection point, making it particularly effective for fitting more complex and variable GP data.

The Burr XII model has four parameters and is well-known for its application in fitting long-tailed distributions, making it useful in a variety of fields such as economics, survival analysis, and reliability^30^. Despite its increased flexibility, the risk of overfitting is higher with the Burr XII model than simpler models, as it has more parameters that need to be estimated. This can result in some parameters becoming statistically insignificant, contributing little to the model fit but increasing complexity. The Inverse paralogistic model, on the other hand, is known for its applications in reliability analysis, particularly for modeling failure times and actuarial loss distributions. It has a moderately flexible structure with fewer parameters, making it less prone to overfitting than the Burr XII, and it is useful for scenarios where GP reaches its peak relatively early. The Log-logistic model is a more straightforward approach, frequently used in survival analysis and biological studies. It is computationally efficient and performs well when data follow predictable pattern. The simplicity also helps to reduce the risk of overfitting.

### Model showdown: consistent and diverging estimates

In examining the estimated and calculated parameters such as maximum $\:m$, $\:{t}_{0.5}$, $\:{GP}_{0.5}$, and $\:{t}^{*}$, the differences between the Burr XII, Inverse paralogistic, and Log-logistic models are relatively minor within feed types. This suggests a degree of generalizability and consistency among the models for these parameters, indicating that any of these models could be reliably used to estimate these aspects of GP dynamics for different types of feeds. However, when looking at other parameters such, as the rate constant $\:r$ and shape parameter $\:a$, the estimated values diverge more significantly among the models, highlighting important variations in how each model interprets and estimates the underlying GP processes.

***Generalizability in estimating*** $\:\varvec{m}$, $\:{\varvec{t}}_{0.5}$, $\:{\varvec{G}\varvec{P}}_{0.5}$, ***and*** $\:{\varvec{t}}^{\varvec{*}}$: Within each feed types, the Burr XII, Inverse paralogistic, and Log-logistic models consistently provided similar estimates for $\:m$, with slight variations. For example, in the Corn category, the estimates for $\:m$ across the models are quite close: 85.9 mL for Inverse paralogistic, 84.4 mL for Burr XII, and 84.3 mL for Log-logistic. This consistency is also observed in other feed types such as Soft cereal and Legumes, with differences typically within 1–2 mL. The relatively small deviations suggest that all three models have strong predictive power for estimating GP potential, making them interchangeable for this parameter. Similar patterns were observed for $\:{t}_{0.5}$, the time at which 50% of GP has occurred. For example, in the Corn category, the $\:{t}_{0.5}$ estimates are 9.05 h for Inverse paralogistic, 8.97 h for Burr XII, and 8.95 h for Log-logistic—demonstrating close agreement between the models. In other feed types like Compound, Soft cereal, and Legumes, the half-life values also remain tightly clustered, typically varying by no more than 0.2–0.3 h. This close alignment across models indicates a shared understanding of the GP curve’s progression and suggests that any of these models could be used to estimate $\:{t}_{0.5}$ with confidence across different feed types. The calculated $\:{GP}_{0.5}\:$values also showed strong alignment between the three models. In the Corn category, the Inverse paralogistic model estimates $\:{GP}_{0.5}$ at 42.97 mL, while the Burr XII and Log-logistic models estimate 42.20 mL and 42.15 mL, respectively. Across other feed types, such as Soft cereal and Legumes, the models again produced comparable estimates (~ 39.0 h), usually within 1 mL of each other. This consistency reinforces the generalizability of these models for estimating $\:{GP}_{0.5}$, suggesting that any of them could serve as a reliable tool for cumulative GP at the half-life stage. The inflection time, $\:{t}^{*}$, is another parameter where the models tended to agree closely. For example, in the Corn category, $\:{t}^{*}$ is estimated at 5.44 h by the Burr XII model, 5.40 h by the Log-logistic model, and 5.30 h by the Inverse paralogistic model. Similar small differences are observed in other feed categories, such as Soft cereal and Legumes, where the estimates typically vary by less than 0.2 h. The high degree of alignment for this parameter suggests that the models share a common approach to estimating the peak fermentation dynamics and GP rates.

#### Divergence in estimating $\:\varvec{r}$ and$\:\varvec{a}$

In contrast to the close alignment for $\:m$, $\:{t}_{0.5}$, $\:{GP}_{0.5}$, and $\:{t}^{*}$, significant differences arise when examining the rate constant $\:r$ and shape parameter $\:a$, which control the speed and curvature of GP. The estimated rate constant $\:r$ for GP exhibited notable variation between the three models. For instance, in the Soft cereal category, the Burr XII model estimated an $\:r$ of 0.255 h⁻¹, while the Inverse paralogistic model estimated 0.253 h⁻¹, and the Log-logistic model showed a much lower value of 0.171 h⁻¹. This pattern of divergence is consistent across other feed types. In the Corn category, for example, the Inverse paralogistic model estimated a rate constant of 0.168 h⁻¹, compared to 0.113 h⁻¹ from the Log-logistic model. The wider range of values for $\:r$ indicates that the models employ different underlying mechanisms to describe the rate of GP.

Similar variability is seen with the shape parameter $\:a$, which affects the curvature and steepness of the GP curve. In the Corn category, for example, the Burr XII model estimated a shape parameter of 2.097, while the Log-logistic model estimated 2.094, and the Inverse paralogistic model produced a lower value of 1.726. These differences were even more pronounced in categories like Legumes, where the Burr XII model estimated an $\:a$ value of 1.970, compared to 1.954 for Log-logistic and 1.662 for Inverse paralogistic. This divergence reflects differing assumptions made by the models about how quickly GP accelerates and decelerates over time, indicating that while the models may be generalizable for estimating cumulative parameters, they vary in their ability to predict the rate at which gas is produced.

Although our findings for GP parameters such as half-life and inflection point generally align with expected trends across feed types (e.g., faster fermentation in cereal and legume categories), we did not attempt direct comparisons with values reported in other studies. This is due to the heterogeneity of datasets, experimental conditions, and the inclusion of novel models in our analysis. Consequently, the emphasis of this study remains on model selection accuracy rather than parameter benchmarking across studies.

### Current study outcome vs. future directions

While this study focused on model selection for GP data analysis, several important aspects should be acknowledged. First, the analysis is based on a large dataset obtained from a specific set of concentrate feed samples representing a relatively wide range. However, this may not fully capture the diversity of feed types and qualities encountered in practice. Therefore, there is ample room for future research to validate these findings across a broader array of feeds and under various experimental conditions.

Second, our focus was primarily on the paradigm of phenomenological models. While these models offer practical benefits, they limit our understanding of the underlying biological processes driving GP. Future studies could investigate the development of hybrid models that combine the flexibility of phenomenological approaches with mechanistic insights.

Additionally, utilizing advanced machine learning techniques, such as neural networks or ensemble methods, could enhance our ability to predict the optimal models for specific feed types or experimental conditions. These approaches can potentially reveal complex relationships between feed characteristics, chemical composition, and GP patterns, leading to more accurate and practical modeling strategies^31^.

Lastly, correlating the parameters of the best-performing models with in vivo digestibility measures could strengthen the link between in vitro GP data and real-world outcomes.

## Conclusion

This study offers a comprehensive evaluation of nonlinear mathematical models for fitting in vitro GP data across different feed types. By systematically selecting and assessing a broad range of models, we identified 21 that performed effectively. It is confirmed that different feed types exhibit distinct GP profiles that can be captured differently by various nonlinear models. Employing machine learning methods that focus on model BIC, we identified Burr XII, Inverse paralogistic, and Log-logistic as the top models, balancing accuracy with simplicity. These three models demonstrated a high level of consistency and generalizability across all data, supporting the hypothesis that a few models can reliably perform well across diverse feed types. This presents a decision-making framework that simplifies the model selection process. Furthermore, the results revealed that model selection had a more significant impact on GP prediction than feed type, highlighting the importance of choosing the right model for accurate GP curve fitting. Our findings may serve as a foundation for the next step in exploring the correlations between the parameters of the best-performing models, feed characteristics, and in vivo digestibility or animal productivity, as this would strengthen the link between research outcomes and livestock farming.

## Data Availability

The datasets and code used in this study are available from the corresponding author upon reasonable request.
